# Community structure of endophytic fungi of four mangrove species in Southern China

**DOI:** 10.1080/21501203.2016.1258439

**Published:** 2016-11-21

**Authors:** Jia-Long Li, Xiang Sun, Liang Chen, Liang-Dong Guo

**Affiliations:** a State Key Laboratory of Mycology, Institute of Microbiology, Chinese Academy of Sciences, Beijing, China; b College of Life Sciences, University of Chinese Academy of Sciences, Beijing, China

**Keywords:** Mangrove, endophytic fungi, diversity, tissue preference, host preference, ITS

## Abstract

Mangrove forests play an important role in subtropical and tropical coastal ecosystems. Endophytic fungi are widely distributed in various ecosystems and have great contribution to global biodiversity. In order to better understand the effects of mangrove species and tissue types on endophytic fungal community, we investigated cultivable endophytic fungi in leaves and twigs of four mangroves *Aegiceras corniculatum, Avicennia marina, Bruguiera gymnorrhiza*, and *Kandelia candel* in Guangxi, China. The four tree species had similar overall colonisation rates of endophytic fungi (24–33%). The colonisation rates of endophytic fungi were higher in twigs (30–58%) than in leaves (6–25%) in the four plant species. A total of 36 endophytic fungal taxa were identified based on morphological characteristics and molecular data, including 35 Ascomycota and 1 Basidiomycota, dominated by *Phomopsis, Phyllosticta, Xylaria, Leptosphaerulina,* and *Pestalotiopsis*. The diversity of endophytic fungi was higher in twigs than in leaves in the four plant species. Some endophytic fungi showed host and tissue preference. The endophytic fungal community composition was different among four mangrove species and between leaf and twig tissues.

## Introduction

Mangroves, situated at the confluence of land and sea in the world’s subtropical and tropical coastal areas, promote the sludge sedimentation and protect coast resident’s life and property from tsunamis (Alongi 2002, 2008; Kathiresan and Rajendran 2005). There are about 9 orders, 20 families, 27 genera, and roughly 70 species of mangrove plants all over the world, occupying a total estimated area of 137,760 km^2^ (Alongi 2002; Giri et al. 2011). Mangrove ecosystem is an important part for near-shore exchanges of nutrients and detritus in geomorphology and hydrodynamics (Ewel et al. 1998; Valiela et al. 2001), provides nursery biotopes for various seabirds and tropical fishes (Kathiresan 2000; Nagelkerken et al. 2000), and harbours high diversity of microorganisms (Kathiresan 2000; Shearer et al. 2007; Cheng et al. 2009; Debbab et al. 2013). The mangrove forests also offer different ecological services and economic goods, like timber, fish, shellfish, fuel, and pharmacy material (Ewel et al. 1998; Alongi 2002), and improve our understanding to the resilience of ecosystem impacted by global climate change (Alongi 2008). However, at least one-third of the world’s mangrove forests have been lost in past 60 years with urban development, aquaculture, mining, and overexploitation (Alongi 2002). Nowadays, approximately 75% of mangroves in world are found in merely 15 countries, and only 6.9% are protected under the existing protected areas network (Giri et al. 2011). Therefore, there is an urgent need of studies aiming to mangrove-associated endophytes.

Endophytic fungi are living within plant organs for some time or whole in their life, without causing apparent harm to their host (Petrini 1991; Sun and Guo 2012). Despite of the extreme living habitats, such as high salinity, low pH, partly anoxic and periodic, soaked by the tide, these salt-tolerant plant species are inhabited by highly diverse endophytic fungi (Suryanarayanan and Kumaresan 2000; Ananda and Sridhar 2002; Shearer et al. 2007; Costa et al. 2012). For example, Suryanarayanan et al. (1998) isolated 39 endophytic fungi from leaves of mangroves *Rhizophora apiculata* and *R. mucronata* in Southern India. Ananda and Sridhar (2002) recovered 35 fungal taxa from roots of mangroves *Acanthus ilicifolius, Avicennia officinalis, R. mucronata*, and *Sonneratia caseolarison* in Western India. Costa et al. (2012) isolated 40 taxa from leaves of mangroves *Avicennia schaueriana, Laguncularia racemosa,* and *Rhizophora mangle* in dry and rainy seasons in Northeast Brazil. Pang et al. (2008) identified 21 fungi from bark, woody, and leaves of *Kandelia candel* in Hong Kong. Xing and Guo (2011) reported 38 taxa from roots and stems of *Ceriops tagal, Rhizophora stylosa, R. apiculata,* and *Bruguiera sexangula* var. *rhynchopetala* on the south coast of China. The mangrove-derived endophytic fungi are a promising source of diverse and structurally unprecedented bioactive natural compounds, which attract considerable attention (Sridhar 2004; Aly et al. 2010; Debbab et al. 2013).

The community structure of mangrove endophytes was affected by the host species, tissue types, and environmental factors (Alias et al. 1995; Suryanarayanan et al. 1998; Kumaresan and Suryanarayanan 2001, 2002; Ananda and Sridhar 2002). For example, Suryanarayanan et al. (1998) found that more endophytic fungi were isolated from leaves of *R. apiculata* and *R. mucronata* in rainy months than in dry period in Southern India. Ananda and Sridhar (2002) reported that the root of *R. mucronata* from the mid-tide level showed greatest number of species of fungi on the west coast of India. Liu et al. (2012) found that the assemblages and dominant species of endophytic *Pestalotiopsis* of mangrove plants varied among host species and geographical locations in South China. Gu et al. (2012) reported that endophytic fungi of *C. tagal* showed certain tissue specificity in Hainan Province, China. Xing et al. (2011) suggested high biodiversity and tissue specificity of endophytic fungi in root, stem, and leaf of *Sonneratia* on the south coast of China.

There are 24 true mangrove species in China, mainly distributed in Hainan, Guangdong, and Guangxi, accounting for 94% of the total mangrove area of China (Chen et al. 2009). Nevertheless, endophytic fungal community of mangroves was poorly understood in China. To better understand the effects of plant species and tissue types on endophytic fungal community, we selected four mangroves in Guangxi, Southern China. The endophytic fungi were isolated from twigs and leaves of mangroves and identified according to morphological characteristics and molecular data. The aim of the present study was to reveal how the colonisation rate, diversity, and community composition of endophytic fungi would differ among plant species and tissue types of the four mangroves. This study provides preliminary data of mangrove endophytic fungi for future studies in bioactive natural products.

## Materials and methods

### Study site and sampling procedure

The study was carried out in the Beilun Estuary National Reserve of Guangxi, South China (21.62°N, 108.23°E). This site has a mean annual temperature of 22.2°C, mean annual precipitation of 2500–2700 mm and soil salinity of 0.5–1.2%. In late April 2014, we selected four mangrove species *Aegiceras corniculatum* (Myrsinaceae), *Avicennia marina* (Verbenaceae), *Bruguiera gymnorrhiza,* and *K. candel* (Rhizophoraceae) in the site. A total of 10 mature individuals of each plant species (except for 9 individuals for *A. marina*) were randomly chosen and the individuals of one plant species were 50 m away from each other. One twig (ca. 0.8 cm in diameter) with attached leaves was collected from each individual tree and immediately placed in plastic bags, labelled, and transported to laboratory. Samples were stored at 4°C and processed within 4 days.

### Isolation and identification of endophytic fungi

The sampling regime was designed with the intention of isolating as many endophytic species as possible from the samples. One twig of each individual tree was cut into 5-mm long segments (ca. 0.8 cm in diameter) and five leaves were removed from one twig and cut into discs (5 mm in diameter). A total of 10 twig segments and 10 leaf discs were randomly selected from each sample. In total, 780 segments were used in this study.

Surface sterilisation followed the method of Guo et al. (2000). Segments were surface sterilised by consecutive immersion for 1 min in 75% ethanol, 3 min in 3.25% sodium hypochlorite, and 30 s in 75% ethanol. Five segments were then evenly placed in each 90 mm Petri dish containing malt extract agar (MEA, 2%). Benzylpenicillin sodium (50 mg/L, North China Pharmaceutical Group Corporation, China) was added to suppress bacterial growth. Petri dishes were sealed, incubated for 2 months at 25°C, and examined periodically. When fungal colonies developed, they were transferred to new Petri dishes with potato dextrose agar (PDA, 2%) for purification. The purified strains were transferred to PDA slants for further study.

Subcultures on PDA were examined periodically and sporulated isolates were identified based on their morphological characteristics, according to Sutton (1980), Ellis (1971, 1976), Seifert et al. (2011) and related references. The non-sporulated cultures were designated as *Mycelia sterilia*, which were divided into different “morphotypes” according to cultural characteristics such as colony colour, texture, and extension rate on MEA (Guo et al. 2000). One representative strain of each morphotype or sporulated isolate was selected for further molecular identification.

### DNA extraction, amplification, sequencing, and identification

Genomic DNA was extracted from fresh cultures following the protocol of Guo et al. (2000). Fresh fungal mycelia (*c*. 50 mg) were scraped from the surface of the agar plate and transferred into a 1.5 mL microcentrifuge tube with 700 µL of preheated (65°C) 2 × CTAB extraction buffer (2% CTAB, 100 mM Tris-HCl, 1.4 M NaCl, 20 mM EDTA, pH 8.0), and *c*. 0.2 g sterilised quartz sand. The mycelium was ground using a glass pestle and then incubated in a 65°C water bath for 30 min with occasional gentle swirling. About 500 mL of phenol:chloroform (1:1) was added into each tube and mixed thoroughly to form an emulsion. The mixture was spun at 12,000 g for 15 min at room temperature in a microcentrifuge and the aqueous phase was transferred into a fresh 1.5 mL tube. The aqueous phase containing DNA was re-extracted with chloroform:isoamyl (24:1) until no interface was visible. About 30 mL of 5 M KOAc was added into the aqueous phase followed by 200 µL of isopropanol and inverted gently to mix. The genomic DNA was precipitated at 9200 g for 2 min in a microcentrifuge. The DNA pellet was washed twice with 70% ethanol and dried using SpeedVad (AES 1010, Savant, Holbrook, NY, USA) for 10 min or until dry.

The internal transcribed spacer (ITS) region of rDNA was amplified using primer pairs ITS4 and ITS1F (White et al. 1990). Amplification was performed in a 50 µL reaction volume which contained polymerase chain reaction (PCR) buffer (20 mM KCl, 10 mM (NH_4_)_2_SO_4_, 2 mM MgCl_2_, 20 mM Tris-HCl, pH 8.4), 200 µm of each deoxyribonucleotide triphosphate, 15 pmols of each primer, *c*. 100 ng template DNA, and 2.5 units of *Taq* polymerase (Biocolor BioScience & Technology Company, Shanghai, China). The thermal cycling programme was as follows: 3 min initial denaturation at 94°C, followed by 35 cycles of 30 s denaturation at 94°C, 30 s annealing at 52°C, and 1 min extension at 72°C; and a final 10 min extension at 72°C. A negative control using water instead of template DNA was included in the amplification process. About 4 mL of PCR products from each PCR reaction were examined by electrophoresis at 80 V for 30 min in a 1% (w/v) agarose gel in 1 × TAE buffer (0.4 M Tris, 50 mM NaOAc, 10 mM EDTA, pH 7.8) and visualised under ultraviolet light after staining with ethidium bromide (0.5 mg/mL). PCR products were directly sequenced with primer pairs as mentioned above in the ABI 3730-XL DNA sequencer (Applied Biosystems, Inc., USA).

A value of 97% ITS identity was used as a DNA barcoding criterion (O’Brien et al. 2005). Sequence-based identifications were made by searching with Blastn in the UNITE+INSD database of fungal nucleotide sequences (Abarenkov et al. 2010; Tedersoo et al. 2014)

### Data analysis

The colonisation rate of endophytic fungi was calculated as the total number of tissue segments infected by fungi divided by the total number of tissue segments incubated (Kumar and Hyde 2004). Since the endophyte colonisation rates did not satisfy homogeneity of variance, non-parametric Kruskal–Wallis test was applied to examine the effects of host identity and tissue type on colonisation rate, followed by using pairwise comparisons at *P* < 0.05. The relative abundance was calculated as the number of isolates of a taxon divided by the total number of isolates of all taxa. Relative frequency was calculated as the total number of plant tissue segments infected by fungi divided by the total number of plant segments incubated (Sun et al. 2011). The importance value (IV) was calculated from the mean of relative frequency and relative abundance, indicated as a percentage (Horton and Bruns 2001; Wang et al. 2010).

Shannon diversity index (*H′*) of endophytic fungi was calculated according to the formula: *H′* = −$\sum_{i=1}^{k}p_{i}\times lnp_{i}$, where *k* is the total number of fungal species, and *p_i_* is the proportion of individuals that species *i* contributes to the total (Pielou 1975). To evaluate the degree of community similarity of endophytic fungi between the plant species, Sorenson’s coefficient (*C*_s_) was employed and calculated according to the following formula: *Cs *= 2*j*/(*a *+ *b*), where *j* is the number of endophytic fungal species recovered from both plant species, *a* is the total number of endophytic fungal species from one plant species, and *b* is the total number of endophytic fungal species from the other plant species (Magurran 2004). Rarefaction curves for observed fungal species richness were calculated using EstimateS Win 9.10 (Colwell and Elsensohn 2014).

## Results

### Colonisation rates of endophytic fungi

**Figure 1.**
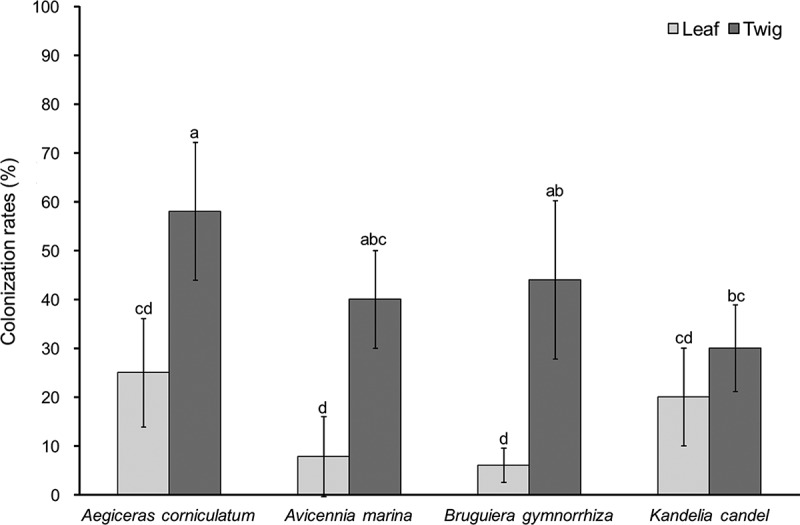
Colonisation rates of endophytic fungi of leaf and twig in four mangrove species. Data are means ± SD. Columns without shared letters denote significant difference at *P* < 0.05.

A total of 301 fungal isolates were recovered from 780 tissue segments of the 4 mangrove species. Of these, 110 isolates were obtained from *A. corniculatum* (28 from leaves and 82 from twigs), 45 from *A. marina* (5 from leaves and 40 from twigs), 83 from *B. gymnorrhiza* (7 from leaves and 76 from twigs), and 63 from *K. candel* (21 from leaves and 42 from twigs) (Table 1). The difference among overall colonisation rates of endophytic fungi in the four hosts was not significant, that is, 33 ± 14% in *A. corniculatum*, 24 ± 12% in *A. marina*, 32 ± 17% in *B. gymnorrhiza*, and 24 ± 10% in *K. candel*. The colonisation rate of endophytic fungi was significantly higher in twigs than in leaves in *A. corniculatum, A. marina,* and *B. gymnorrhiza*, but not in *K. candel* (Figure 1). The fungal colonisation rate from high to low was *A. corniculatum* (58%) > *B. gymnorrhiza* (44%) > *A. marina* (40%) > *K. candel* (30%) in twigs, and *A. corniculatum* (25%) > *K. candel* (20%) > *B. gymnorrhiza* (7.78%) > *A. marina* (6%) in leaves (Figure 1).

**Table 1.** Shannon diversity index (*Hʹ*) and species richness of endophytic fungi isolated from leaves and twigs of the four mangrove species.

	*Aegiceras corniculatum*			*Avicennia marina*			*Bruguiera gymnorrhiza*			*Kandelia candel*		
	Leaf	Twig	Total	Leaf	Twig	Total	Leaf	Twig	Total	Leaf	Twig	Total
No. of samples	100	100	200	90	90	180	100	100	200	100	100	200
No. of infected samples	25	58	83	7	36	43	6	44	50	20	30	50
No. of isolates recovered	28	82	110	5	40	45	7	76	83	21	42	63
*Hʹ*	2.23	2.38	2.69	0.67	2.46	2.51	1.75	2.32	2.45	1.13	2.09	2.31
Richness	12	17	24	2	16	16	6	16	18	6	12	16

### Endophytic fungal diversity

**Figure 2.**
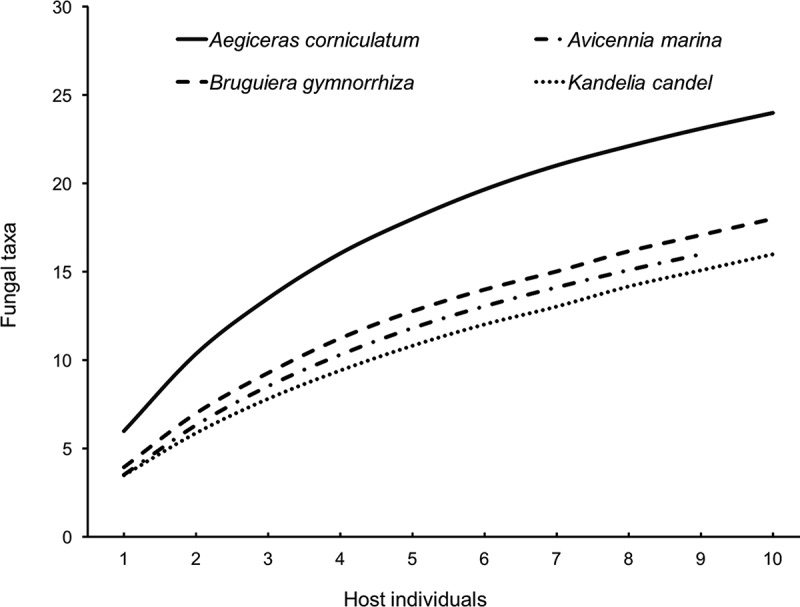
Rarefaction curves for observed endophytic fungi of four mangrove species.

In total, 36 fungal taxa were identified based on morphological characteristics and ITS sequence data. Of the 36 taxa, one belonged to Basidiomycota (*Coprinopsis atramentaria*), and 35 were members of Ascomycota (Table 2). About 24 endophytic taxa were recovered from *A. corniculatum* (12 from leaves and 17 from twigs), 16 from *A. marina* (2 from leaves and 16 from twigs), 18 from *B. gymnorrhiza* (6 from leaves and 16 from twigs), and 16 from *K. candel* (6 from leaves and 12 from twigs). The Shannon diversity index (*H′*) of endophytic fungi from high to low was *A. corniculatum* (2.69) > *A. marina* (2.51) > *B. gymnorrhiza* (2.45) > *K. candel* (2.31) in whole plant, *A. marina* (2.46) > *A. corniculatum* (2.38) > *B. gymnorrhiza* (2.32) > *K. candel* (2.09) in twigs, and *A. corniculatum* (2.23) > *B. gymnorrhiza* (1.75) > *K. candel* (1.13) > *A. marina* (0.67) in leaves (Table 1). The Sorenson’s similarity coefficient analysis showed low similarity of endophytic fungal community (*Cs*: 0.38–0.59) among the four mangrove species (Table 3). Species accumulation curves demonstrated that the observed fungal richness continuously rose in all four host plants, suggesting that further sampling would recover more endophytic taxa (Figure 2).

**Table 2.** Molecular identification of isolated endophytic fungi based on ITS sequences.

Fungal taxa	GenBank accession no.	Closest blast match (GenBank accession no.)	Similarity (%)
*Alternaria alternata*	KX065252	*Alternaria alternata* (KM580660)	100
*Cladosporium perangustum*	KX065253	*Cladosporium perangustum* (KM485631)	100
*Coprinopsis atramentaria*	KX065254	*Coprinopsis atramentaria* (KJ817302)	99
*Creosphaeria sassafras*	KX065255	*Creosphaeria sassafras* (KJ572192)	100
*Dothideomycetessp.*	KX065256	*Dothideomycetessp*. (JQ905828)	100
*Exophiala oligosperma*	KX065257	*Exophiala oligosperma* (LC018821)	100
*Fusarium equiseti*	KX065258	*Fusarium equiseti* (KP068925)	100
*Fusarium striatum*	KX065259	*Fusarium striatum* (KM231798)	100
*Guignardiasp.*	KX065260	*Guignardia ardisiae* (AB454283)	95
*Hortaea werneckii*	KX065261	*Hortaea werneckii* (JX177611)	100
*Hypoxylon investiens*	KX065262	*Hypoxylon investiens* (JN979428)	99
*Leptosphaerulina chartarum*	KX065263	*Leptosphaerulina chartarum* (GQ254687)	99
*Leptosphaerulinasp.*	KX065264	*Leptosphaerulina chartarum* (GQ254687)	96
*Lophiostomasp.*	KX065265	*Lophiostomasp*. (GQ254683)	99
*Mycosphaerellasp.*	KX065266	*Mycosphaerellasp*. (HQ731642)	99
*Neofusicoccum australe*	KX065267	*Neofusicoccum australe* (FJ441624)	100
*Neofusicoccum parvum*	KX065268	*Neofusicoccum parvum* (KJ193665)	100
*Paraconiothyrium archidendri*	KX065269	*Paraconiothyrium archidendri* (JX496049)	98
*Passalorasp.*	KX065270	*Passalorasp*. (GU214642)	99
*Pestalotiopsis humus*	KX065271	*Pestalotiopsis humus* (KM199319)	100
*Phomopsis azadirachtae*	KX065272	*Phomopsis azadirachtae* (KJ427813)	100
*Phomopsissp*.1	KX065273	*Phomopsissp*. (GU066708)	99
*Phomopsissp*.2	KX065274	*Phomopsissp*. (FJ527874)	99
*Phomopsissp*.3	KX065275	*Phomopsissp*. (FJ037768)	100
*Phomopsissp*.4	KX065276	*Phomopsissp*. (FJ037761)	100
*Phomopsissp*.5	KX065277	*Phomopsissp*. (EU236706)	100
*Phyllosticta capitalensis*	KX065278	*Phyllosticta capitalensis* (LM994823)	100
*Phyllostictasp.*	KX065279	*Phyllosticta aristolochiicola* (JX486129)	95
*Pseudoplectaniasp.*	KX065280	*Pseudoplectania ericae* (KF305721)	90
*Pyronemasp.*	KX065281	*Pyronemasp*. (HQ829058)	100
*Rhizopycnissp.*	KX065282	*Rhizopycnissp*. (JN198469)	98
*Sordariomycetessp.*	KX065283	*Sordariomycetessp*. (JQ761583)	100
*Sporormiaceaesp.*	KX065284	*Amorosia littoralis* (AM292047)	87
*Xylaria feejeensis*	KX065285	*Xylaria feejeensis* (KJ767110)	100
*Xylariasp*.1	KX065286	*Xylariasp*. (AB701348)	99
*Xylariasp*.2	KX065287	*Xylaria bambusicola* (JX256820)	91

**Table 3.** Sorenson’s similarity coefficients (*Cs*) of endophytic fungal community between four mangroves.

Host species	*Aegiceras corniculatum*	*Avicennia marina*	*Bruguiera gymnorrhiza*
*A. marina*	0.50		
*B. gymnorrhiza*	0.48	0.41	
*Kandelia candel*	0.55	0.38	0.59

### Endophytic fungal community composition

**Figure 3.**
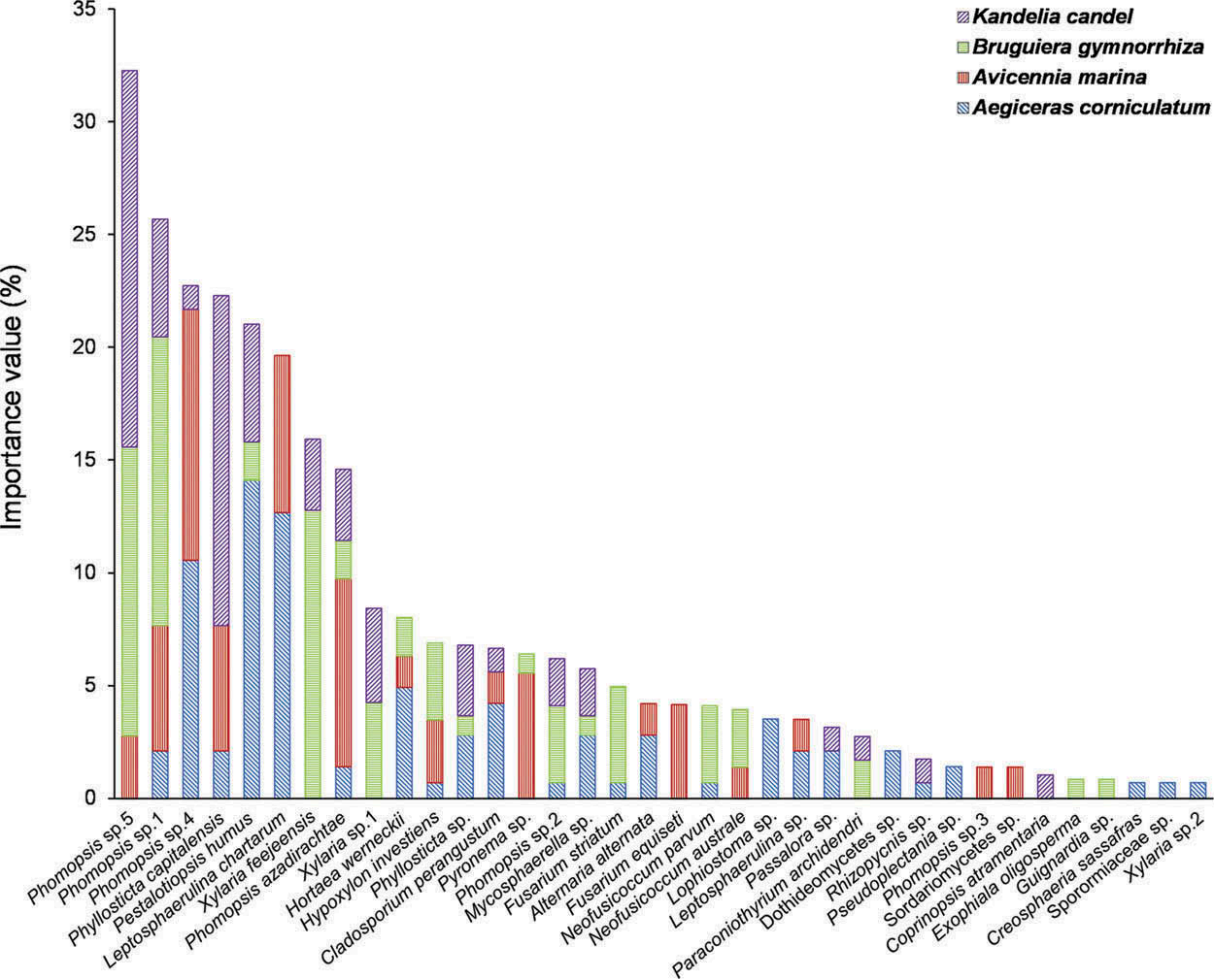
Importance value of endophytic fungi in four mangrove species.

**Figure 4.**
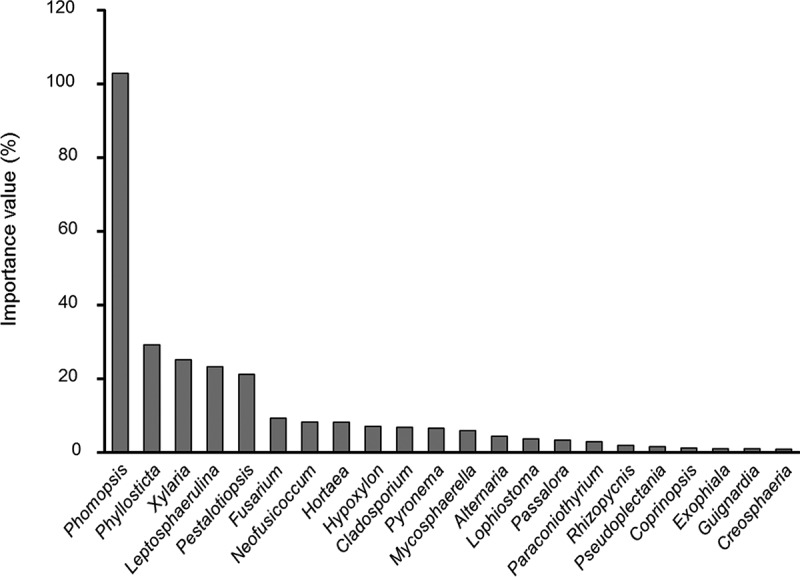
Importance value of genera of endophytic fungi.

**Figure 5.**
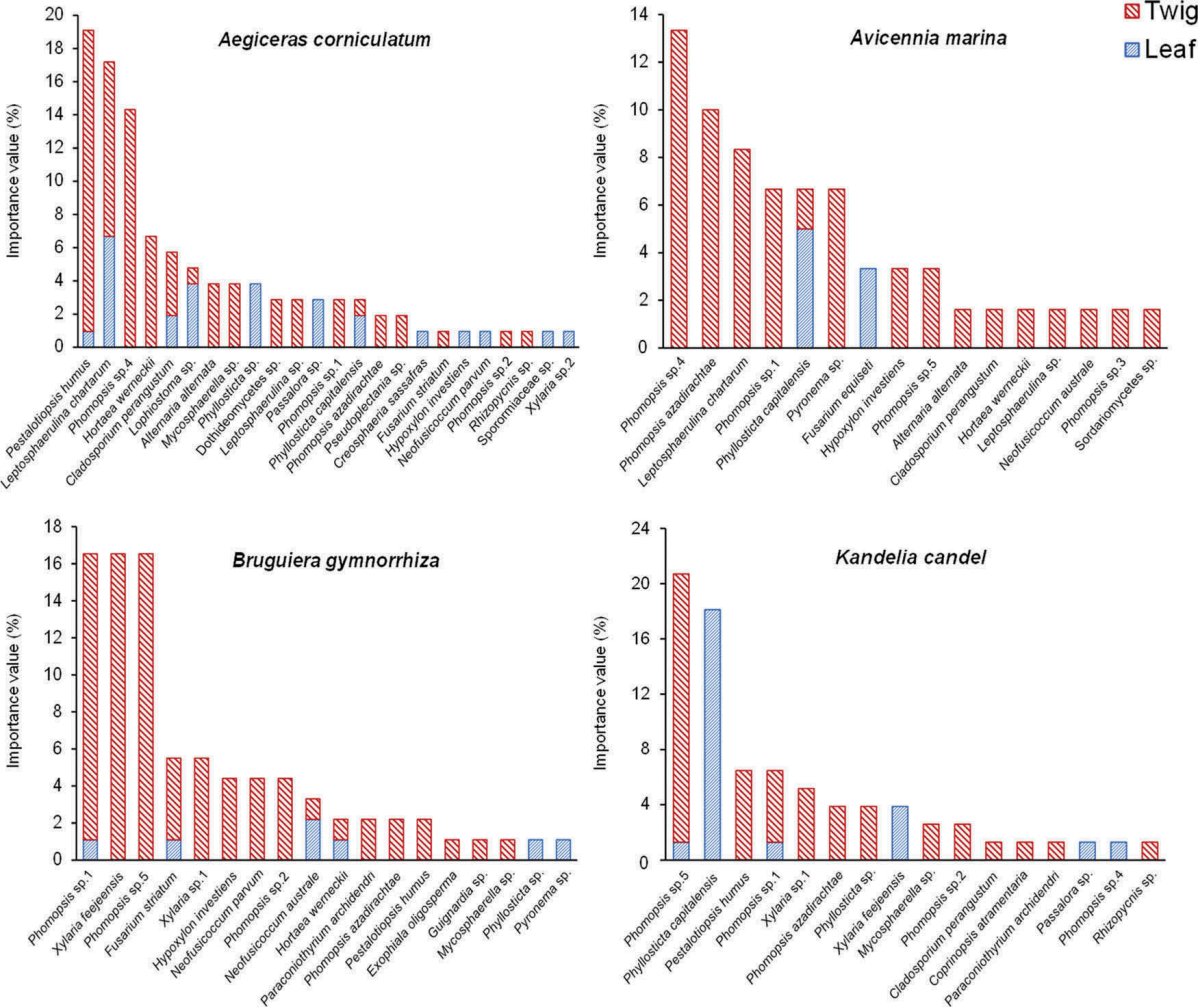
Importance value of endophytic fungi in twig and leaf in each mangrove species.

The four mangrove plants harboured 13 abundant endophytic fungi (IV ≥ 5) and 23 rare taxa (IV < 5) (Figure 3). *Phomopsis, Phyllosticta, Xylaria, Leptosphaerulina,* and *Pestalotiopsis* were dominant in current study (Figure 4). More endophytic taxa and isolates were recovered in twigs than in leaves in the four mangrove species (Figure 5).

Among 13 abundant endophytic taxa, *Pestalotiopsis humus, Leptosphaerulina chartarum, Phomopsis sp*.4, *Hortaea werneckii,* and *Cladosporium perangustum* were abundant in plant *A. corniculatum* and were mainly recovered from twigs, while *L. chartarum* distributed evenly in twigs and leaves (Figure 5). In plant *A. marina*, the abundant fungi *L. chartarum, Phomopsis azadirachtae, Phomopsis sp*.1, *Phomopsis sp*.4, and *Pyronema sp*. were recovered exclusively in twigs, but *Phyllosticta capitalensis* was mainly distributed in leaves (Figure 5). In plant *B. gymnorrhiza*, the abundant fungi *Fusarium striatum, Phomopsis sp*.1, *Phomopsis sp*.5, *Xylaria feejeensis,* and *Xylaria sp*.1 were mainly distributed in twigs (Figure 5). In plant *K. candel*, the abundant fungi *Phomopsis sp*.5, *P. humus, Phomopsis sp*.1, and *Xylaria sp*.1 were mainly distributed in twigs, but *P. capitalensis* was only isolated from leaves (Figure 5).

## Discussion

### Effects of plant identity and tissue type on colonisation rate of endophytic fungi

The overall colonisation rate of endophytic fungi was similar in the four mangroves (24–33%) in this study. This result is in consistence with previous studies on mangrove endophytes (Gilbert et al. 2002; Deng et al. 2010). Furthermore, we found that the colonisation rate of endophytic fungi was higher in twigs than in leaves in the four hosts. Similar results were reported in previous studies of mangrove plants (Pang et al. 2008; Xing et al. 2011) and non-mangrove plants (Fisher et al. 1994; Wang and Guo 2007; Sun et al. 2011). For example, Xing et al. (2011) found that the colonisation rate of endophytic fungi was significantly higher in stem than in leaf in four mangrove *Sonneratia* species at the south coast of China. De Souza Sebastianes et al. (2013) reported that the colonisation rate of endophytic fungi was significantly higher in branch than in leaf among three mangrove species in Brazil. The possible explanation might be that the structure and substrates are different between twig and leaf tissues, or that the twigs are more permanent than leaves, which influence the colonisation of endophytic fungi (Taylor et al. 1999; Gilbert et al. 2002; Wang and Guo 2007; Guo et al. 2008).

### Effects of plant identity on endophytic fungal community

We found that *Phomopsis, Phyllosticta, Xylaria, Leptosphaerulina,* and *Pestalotiopsis* were dominant in the four mangroves, as reported in some previous studies (Bayman et al. 1998; Suryanarayanan and Kumaresan 2000; Chaeprasert et al. 2010; Xing and Guo 2011). For example, *Phomopsis* fungi were widely isolated from mangroves (Suryanarayanan et al. 1998; Suryanarayanan and Kumaresan 2000; Pang et al. 2008; Xing et al. 2011; Xing and Guo 2011) and non-mangrove plants (Taylor et al. 1999; Guo et al. 2000, 2008; Cannon and Simmons 2002; Murali et al. 2006; Sun et al. 2011, 2012). In addition, *Pestalotiopsis* as generalist fungi were common mangrove endophytes and widely occurred on plants across Palmae, Rhizophoraceae, Planchonellae, and Podocarpaceae in China (Liu et al. 2010a, 2012; Xing and Guo 2011; Gong et al. 2014). *Xylaria*, as endophytic fungi were dominant in subtropical and tropical ecosystems (Bayman et al. 1998; Guo et al. 2000). *Phyllosticta* taxa were the most frequently isolated fungi of 10 mangrove plants in Thailand (Chaeprasert et al. (2010). In addition, *L. chartarum* as the predominant species was first reported in mangrove species in this study.

The community composition of endophytic fungi was different in the four mangroves in our study, which was consistent with previous studies that endophytic fungal community composition was conspicuously affected by mangrove species (Suryanarayanan et al. 1998; Kumaresan and Suryanarayanan 2001; Ananda and Sridhar 2002; Arfi et al. 2012; Costa et al. 2012; Liu et al. 2012). Furthermore, we found that some abundant endophytic fungi were different in the four mangroves. For example, *H. werneckii* and *C. perangustum* were obtained only from plant *A. corniculatum, P. azadirachtae,* and *Pyronema sp*. from plant *A. marina*, and *F. striatum* and *X. feejeensis* from plant *B. gymnorrhiza*. In addition, some abundant fungi were found in two plant species in this study. For example, *P. humus* was obtained from *A. corniculatum* and *K. candel. Phomopsis sp*.1 was recovered from *A. marina* and *B. gymnorrhiza*. Similarly, previous studies have shown that some endophytic fungi were abundant in certain mangroves (Pang et al. 2008; Chaeprasert et al. 2010; Liu et al. 2010b; Chen et al. 2012; Gong et al. 2014). For example, Gong et al. (2014) concluded that *Pestalotiopsis* was dominant in *A. corniculatum* in Guangxi Province, China. Xing and Guo (2011) found that *Pestalotiopsis* along with *Phomopsis* were the most frequent endophytes in four Rhizophoraceae mangrove species on the south coast of China. Chaeprasert et al. (2010) suggested *Phyllosticta* was the most frequently isolated fungus from *Avicennia alba*. Pang et al. (2008) reported that *Xylaria sp*. was dominant endophytic species in *K. candel*. These results suggested that some endophytic fungi show certain host preference.

### Effects of tissue type on endophytic fungal community

We found that the diversity of endophytic fungi was higher in twigs than in leaves in the four plant species. Similar results were reported in previous studies (Maria and Sridhar 2003; Wang and Guo 2007; Deng et al. 2010; Sun et al. 2012). For example, Xing et al. (2011) reported more fungal endophytes in twigs than in leaves of four mangroves on the south coast of China. Pang et al. (2008) found a higher number of fungal endophytes in bark than in leaves of mangrove *K. candel* in Hong Kong. De Souza Sebastianes et al. (2013) reported more endophytic fungal taxa in twigs than in leaves of mangroves *R. mangle, A. schaueriana,* and *L. racemosa* in Brazil.

We found that the community composition of endophytic fungi was different between twigs and leaves in the four mangroves. Similar results have been shown by previous studies (Ananda and Sridhar 2004; Pang et al. 2008; De Souza Sebastianes et al. 2013; Gong et al. 2014). Furthermore, we found that some abundant endophytic fungi were different in leaf and twig tissues. For example, five *Phomopsis* fungi were much more abundant in twigs than in leaves in this study. Similarly, Xing et al. (2011) found that *Phomopsis* species more frequently distributed in stems than in leaves in *Sonneratia apetala, S. hainanensis* and *S. paracaseolaris* on southern coast of China. *Pestalotiopsis* was much abundant in twigs rather than in leaves in this study, which was consistent with previous studies in mangrove plants (Pang et al. 2008; Liu et al. 2010b; Xing et al. 2011; Gong et al. 2014), and was broadly distributed in Southern China, occurring on a wide range of host (Liu et al. 2007; Wei et al. 2007). *Phyllosticta* fungi were abundant in leaves of the four mangrove trees, as reported in previous mangrove studies (Suryanarayanan et al. 1998; Suryanarayanan and Kumaresan 2000; Chaeprasert et al. 2010). *L. chartarum* and *X. feejeensis* were isolated only from twigs of *A. marina* and *B. gymnorrhiza*, respectively. These results suggested that some endophytic fungi showed certain tissue preference (Guo et al. 2008; Xing et al. 2011; Sun et al. 2012).

## Conclusion

Our study investigated the endophytic fungal community associated with twigs and leaves among four mangrove species in Southern China. Although the overall colonisation rate of endophytic fungi was similar among four investigated mangrove plants, the colonisation rate of endophytic fungi was higher in twigs than in leaves. A total of 36 endophytic fungal taxa were identified according to morphological characteristics and ITS sequences, dominated by *Phomopsis, Phyllosticta, Xylaria, Leptosphaerulina,* and *Pestalotiopsis*. The diversity of endophytic fungi was higher in twigs than in leaves among the four mangrove species. Some endophytic fungi showed certain host and tissue preference. The community composition of endophytic fungi was different among host species and between tissue types.
